# Supplementary material for: The citrate transporters SLC13A5 and SLC25A1 elicit different metabolic responses and phenotypes in the mouse
Source: Commun Biol. 2023 Sep 9;6:926. doi: 10.1038/s42003-023-05311-1 (PMC10492862; doi:10.1038/s42003-023-05311-1)
Supplement: Supplementary file 2 — Supplementary Information [file 42003_2023_5311_MOESM2_ESM.pdf]

Supplementary Figures

The citrate transporters SLC13A5 and SLC25A1 elicit different metabolic responses and phenotypes in the mouse

Gonzalo Fernandez-Fuente[1,2], Katherine A. Overmyer[3,4], Alexis J. Lawton[3,5], Ildiko Kasza[6], Samantha L. Shapiro[1,2], Patricia Gallego-Muñoz[7], Joshua J. Coon[3,4,8], John M. Denu[3,5], Caroline M. Alexander[6], Luigi Puglielli[1,2,9,10,*]

[1]Department of Medicine, University of Wisconsin-Madison, Madison, WI, USA
[2]Waisman Center, University of Wisconsin-Madison, Madison, WI, USA
[3]Department of Biomolecular Chemistry, University of Wisconsin-Madison, Madison, WI, USA
[4]Morgridge Institute for Research, Madison, WI, USA
[5]Wisconsin Institute for Discovery, University of Wisconsin-Madison, Madison, WI, USA
[6]McArdle Laboratory for Cancer Research, University of Wisconsin-Madison, Madison, WI, USA
[7]Department of Cell Biology, Genetics, Histology and Pharmacology, Faculty of Medicine, University of Valladolid, Valladolid, Spain
[8]Department of Chemistry, University of Wisconsin-Madison, Madison, WI, USA
[9]Geriatric Research Education Clinical Center, Veterans Affairs Medical Center, Madison, WI, USA
[10]Department of Neuroscience, University of Wisconsin-Madison, Madison, WI, USA

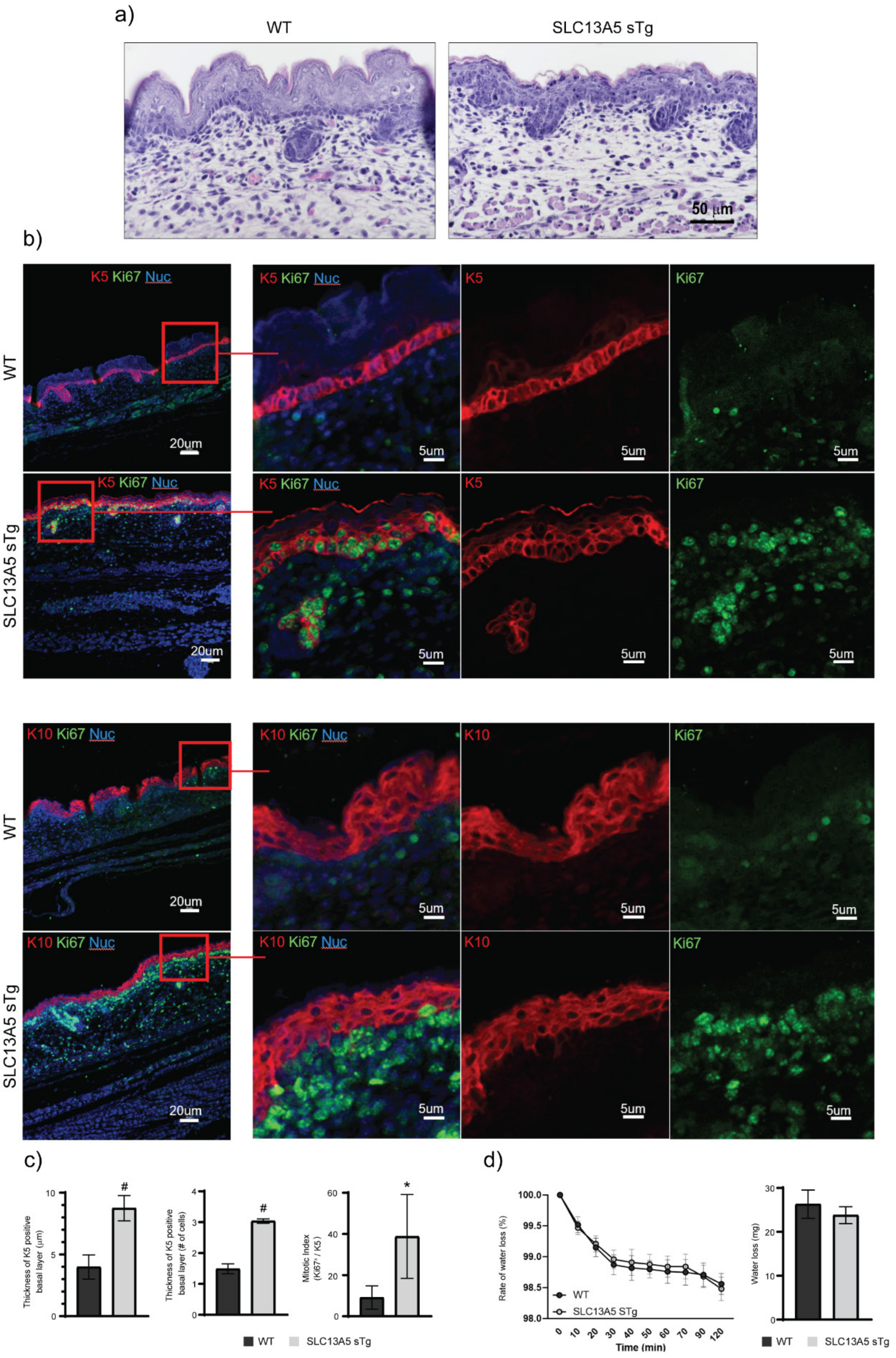
a)
WT
SLC13A5 sTg
50 μm
b)
K5 Ki67 Nuc
K5
Ki67
WT
SLC13A5 sTg
20um
5um
K10 Ki67 Nuc
K10
c)
Thickness of K5 positive basal layer (μm)
Thickness of K5 positive basal layer (# of cells)
Mitotic Index (Ki67⁺ / K5)
#
*
WT
SLC13A5 sTg
d)
Rate of water loss (%)
Time (min)
WT
SLC13A5 STg
Water loss (mg)

Supplementary Figure 1. SLC13A5 sTg^{OC} mice display skin defects before birth (E18).

(a) Representative H&E staining of skin sections from WT and SLC13A5 sTg^{OC} embryos.

(b) Immunostaining of 5μm paraffin-embedded skin slices from WT and SLC13A5 sTg^{OC} embryos. Keratin 5 (upper panel) and Keratin 10 (lower panel) combined with proliferation marker Ki67 and nuclear staining.

(c) Keratin 5 basal layer thickness (μm and number of cells) and Mitotic index (n=3/group). **P*<0.05, $^{\#}P$<0.0005 via mean comparison using Student's t-test.

(d) Water loss expressed as percentage rate over time and total milligrams (n=8 WT; n=5 SLC13A5 sTg mice).

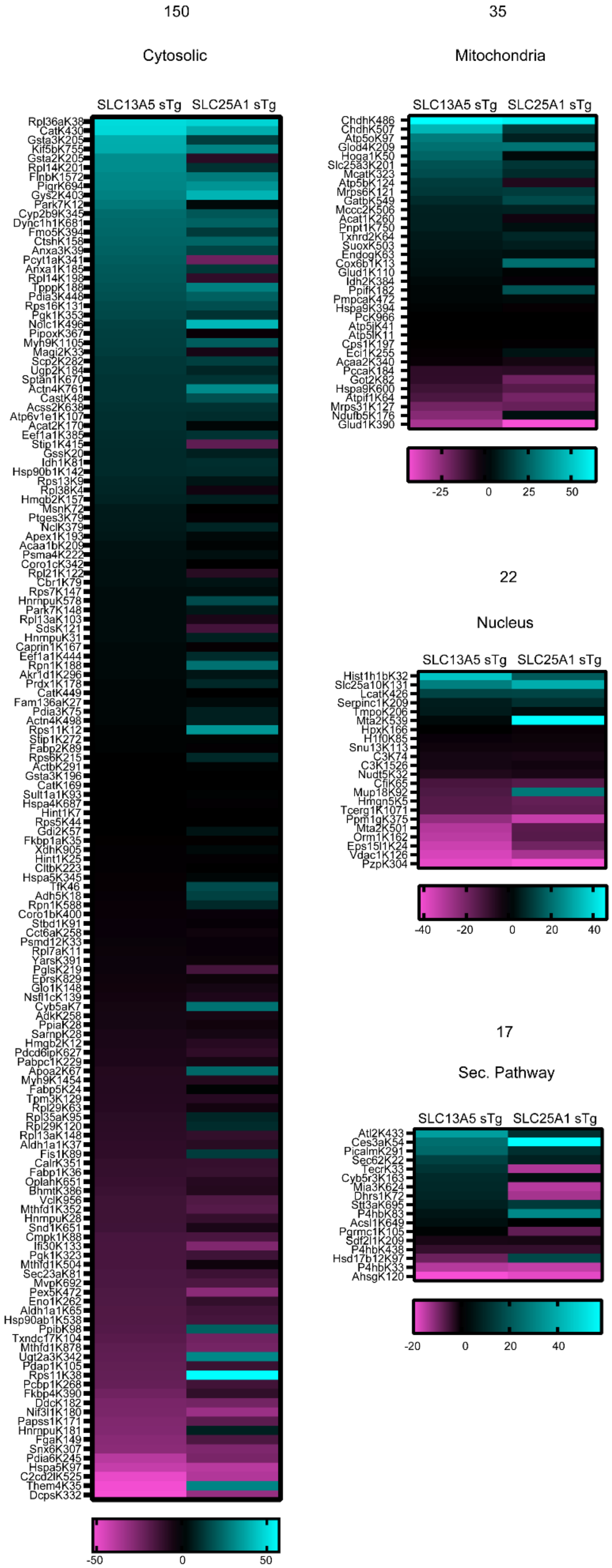
150
Cytosolic
SLC13A5 sTg
SLC25A1 sTg
RpI36aK38
CatK430
Gsta3K205
Kif5bK755
Gsta2K205
RpI14K201
FlnbK1572
PigrK694
Gys2K403
Park7K12
Cyp2b9K345
Dync1h1K681
Fmo5K394
CtshK158
Anxa3K39
Pcyt1aK341
Anxa1K185
RpI14K198
TpppK188
Pdia3K448
Rps16K131
Pgk1K353
Nolc1K496
PipoxK367
Myh9K1105
Magi2K33
Scp2K282
Ugp2K184
Sptan1K670
Actn4K761
CastK48
Acss2K638
Atp6v1e1K107
Acat2K170
Eef1a1K385
Stip1K415
GssK20
Idh1K81
Hsp90b1K142
Rps13K9
RpI38K4
Hmgb2K157
MsnK72
Ptges3K79
NclK379
Apex1K193
Acaa1bK209
Psma4K222
Coro1cK342
RpI21K122
Cbr1K79
Rps7K147
HnrnpuK578
Park7K148
RpI13aK103
SdsK121
HnrnpuK31
Caprin1K167
Eef1a1K444
Rpn1K188
Akr1d1K296
Prdx1K178
CatK449
Fam136aK27
Pdia3K75
Actn4K498
Rps11K12
Stip1K272
Fabp2K89
Rps6K215
ActbK291
Gsta3K196
CatK169
Sult1a1K93
Hspa4K687
Hint1K7
Rps5K44
Gdi2K57
Fkbp1aK35
XdhK905
Hint1K25
CltbK223
Hspa5K345
TfK46
Adh5K18
Rpn1K588
Coro1bK400
Stbd1K91
Cct6aK258
Psmd12K33
RpI7aK11
YarsK391
PglsK219
EprsK829
Glo1K148
Nsfl1cK139
Cyb5aK7
AdkK258
PpiaK28
SarnpK28
Hmgb2K12
Pdcd6ipK627
Pabpc1K229
Apoa2K67
Myh9K1454
Fabp5K24
Tpm3K129
RpI29K63
RpI35aK95
RpI29K120
RpI13aK148
Aldh1a1K37
Fis1K89
CalrK351
Fabp1K36
OplahK651
BhmtK386
VclK956
Mthfd1K352
HnrnpuK28
Snd1K651
Cmpk1K88
Ifi30K133
Pgk1K323
Mthfd1K504
Sec23aK81
MvpK692
Pex5K472
Eno1K262
Aldh1a1K65
Hsp90ab1K538
PpibK98
Txndc17K104
Mthfd1K878
Ugt2a3K342
Pdap1K105
Rps11K38
Pcbp1K268
Fkbp4K390
DdcK182
Nif3l1K180
Papss1K171
HnrnpuK181
FgaK149
Snx6K307
Pdia6K245
Hspa5K97
C2cd2lK525
Them4K35
DcpsK332
-50
0
50
35
Mitochondria
SLC13A5 sTg
SLC25A1 sTg
ChdhK486
ChdhK507
Atp5oK97
Glod4K209
Hoga1K50
Slc25a3K201
McatK323
Atp5bK124
Mrps6K121
GatbK549
Mccc2K506
Acat1K260
Pnpt1K750
Txnrd2K64
SuoxK503
EndogK63
Cox6b1K13
Glud1K110
Idh2K384
PpifK182
PmpcaK472
Hspa9K394
PcK966
Atp5jK41
Atp5lK11
Cps1K197
Eci1K255
Acaa2K340
PccaK184
Got2K82
Hspa9K600
Atpif1K64
Mrps31K127
Ndufb5K176
Glud1K390
-25
0
25
50
22
Nucleus
SLC13A5 sTg
SLC25A1 sTg
Hist1h1bK32
Slc25a10K131
LcatK426
Serpinc1K209
TmpoK206
Mta2K539
HpxK166
H1f0K85
Snu13K113
C3K74
C3K1526
Nudt5K32
CfiK65
Mup18K92
Hmgn5K5
Tcerg1K1071
Ppm1gK375
Mta2K501
Orm1K162
Eps15l1K24
Vdac1K126
PzpK304
-40
-20
0
20
40
17
Sec. Pathway
SLC13A5 sTg
SLC25A1 sTg
Atl2K433
Ces3aK54
PicalmK291
Sec62K22
TecrK33
Cyb5r3K163
Mia3K624
Dhrs1K72
Stt3aK695
P4hbK83
Acsl1K649
Pgrmc1K105
Sdf2l1K208
P4hbK438
Hsd17b12K97
P4hbK33
AhsgK120
-20
0
20
40

Supplementary Figure 2. Heat-maps showing the profile of the 224 overlapping acetylation sites among the cytosolic, mitochondria, nucleus, and secretory pathway fractions found in both SLC13A5 sTg and SLC25A1 sTg mice, as compared to WT littermates. Mice were 2 month-old when analyzed.

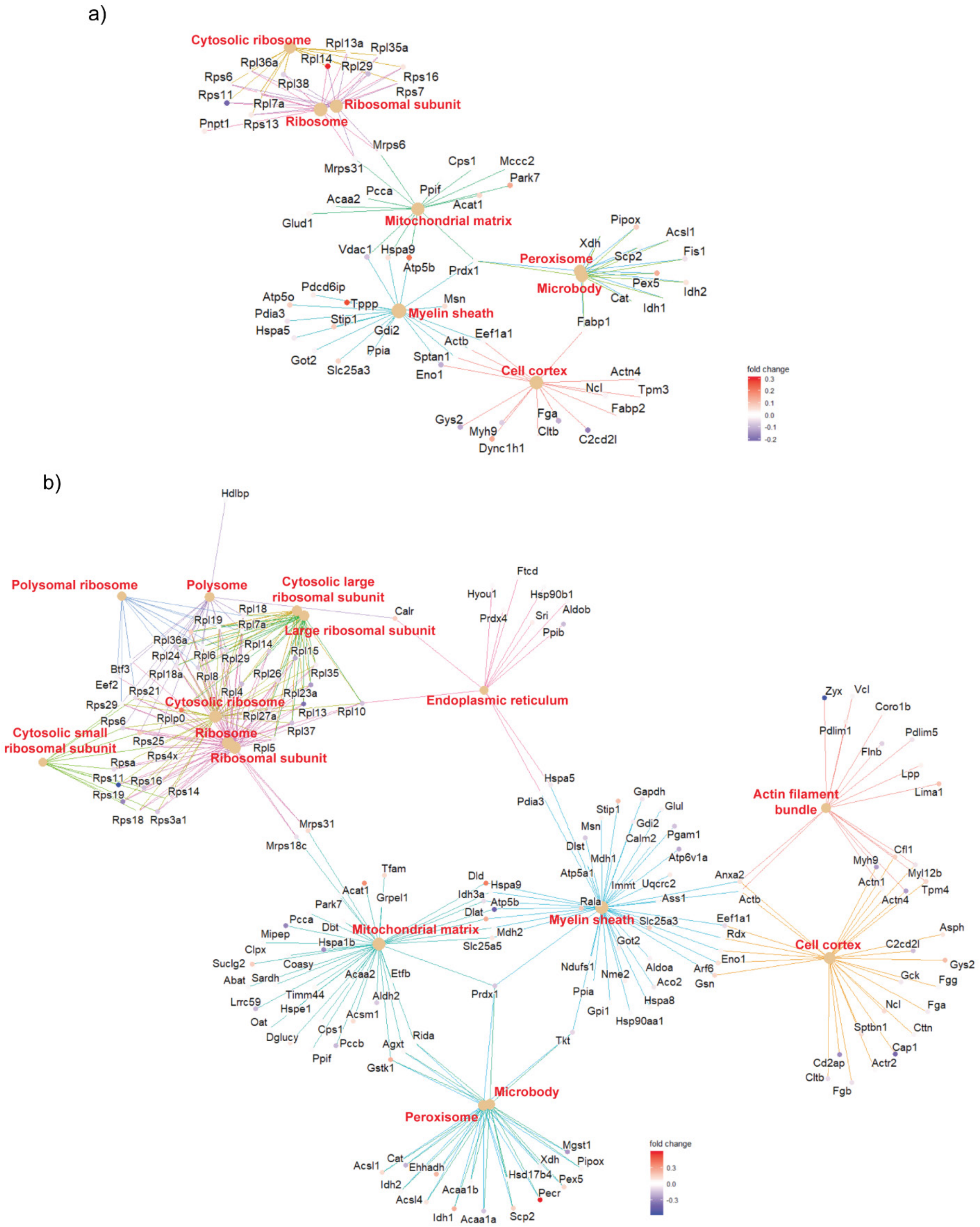
a)
Cytosolic ribosome
Rpl13a
Rpl35a
Rpl36a
Rpl14
Rpl29
Rps6
Rpl38
Rps16
Rps11
Rps7
Rpl7a
Ribosomal subunit
Pnpt1
Rps13
Ribosome
Mrps6
Mrps31
Cps1
Mccc2
Park7
Pcca
Ppif
Acaa2
Acat1
Glud1
Mitochondrial matrix
Pipox
Acsl1
Xdh
Fis1
Vdac1
Hspa9
Peroxisome
Scp2
Atp5b
Prdx1
Microbody
Pex5
Idh2
Pdcd6ip
Cat
Atp5o
Tppp
Idh1
Msn
Pdia3
Stip1
Myelin sheath
Fabp1
Hspa5
Gdi2
Eef1a1
Actb
Ppia
Got2
Sptan1
Slc25a3
Eno1
Cell cortex
Actn4
Ncl
Tpm3
Fabp2
Gys2
Fga
Myh9
Cltb
C2cd2l
Dync1h1
fold change
0.3
0.2
0.1
0.0
-0.1
-0.2
b)
Hdlbp
Ftcd
Hyou1
Hsp90b1
Polysomal ribosome
Polysome
Cytosolic large ribosomal subunit
Prdx4
Sri
Aldob
Calr
Ppib
Rpl18
Rpl19
Rpl7a
Large ribosomal subunit
Rpl36a
Rpl14
Rpl24
Rpl6
Rpl29
Rpl15
Btf3
Rpl18a
Rpl8
Rpl26
Rpl35
Eef2
Rps21
Rpl4
Rpl23a
Rps29
Cytosolic ribosome
Rpl13
Rpl10
Endoplasmic reticulum
Rps6
Rplp0
Rpl27a
Cytosolic small ribosomal subunit
Rps25
Ribosome
Rpl37
Rpl5
Rps4x
Ribosomal subunit
Rpsa
Rps11
Rps16
Rps19
Rps14
Rps18
Rps3a1
Zyx
Vcl
Coro1b
Pdlim1
Pdlim5
Flnb
Lpp
Hspa5
Lima1
Pdia3
Gapdh
Actin filament bundle
Stip1
Glul
Gdi2
Msn
Calm2
Pgam1
Dlst
Mrps31
Mdh1
Atp6v1a
Mrps18c
Cfl1
Tfam
Atp5a1
Myh9
Immt
Uqcrc2
Myl12b
Dld
Anxa2
Actn1
Acat1
Hspa9
Tpm4
Grpel1
Idh3a
Rala
Ass1
Actb
Actn4
Park7
Atp5b
Pcca
Dlat
Myelin sheath
Slc25a3
Eef1a1
Dbt
Asph
Mipep
Mitochondrial matrix
Mdh2
Rdx
Hspa1b
Cell cortex
Clpx
Slc25a5
C2cd2l
Coasy
Suclg2
Eno1
Gys2
Ndufs1
Arf6
Abat
Sardh
Acaa2
Etfb
Aldoa
Nme2
Gck
Fgg
Gsn
Aco2
Lrrc59
Timm44
Aldh2
Prdx1
Ppia
Hspa8
Ncl
Fga
Hspe1
Oat
Acsm1
Gpi1
Hsp90aa1
Sptbn1
Cttn
Cps1
Rida
Dglucy
Pccb
Agxt
Tkt
Cap1
Cd2ap
Actr2
Ppif
Gstk1
Cltb
Fgb
Microbody
Peroxisome
Mgst1
Cat
Xdh
Pipox
Acsl1
Ehhadh
Hsd17b4
Pex5
Idh2
Acaa1b
Pecr
Acsl4
Idh1
Acaa1a
Scp2
fold change
0.3
0.0
-0.3

Supplementary Figure 3. STRING analysis of unique changes in stoichiometry of lysine acetylation.

(a) Gene-network plot of proteins (total of 193) harboring the acetylation sites that were significantly changed from WT and shared by SLC13A5 and SLC25A1 sTg mice. Plot constructed via an overrepresentation analysis using the GO cellular component function database. The color of each dot indicates the fold change in the stoichiometry of lysine acetylation. The top 8 categories sorted by enrichment score are shown with a filtered FDR score of 0.05.

(b) Gene-network plot of proteins (total of 500) harboring the acetylation sites that were significantly changed in SLC13A5 mice, as compared to SLC25A1 sTg mice. Plot constructed via an overrepresentation analysis using the GO cellular component function database. The color of each dot indicates the fold change in the stoichiometry of lysine acetylation. The top 15 categories sorted by enrichment score are shown with a filtered FDR score of 0.05.

Mice were 2 month-old when analyzed.

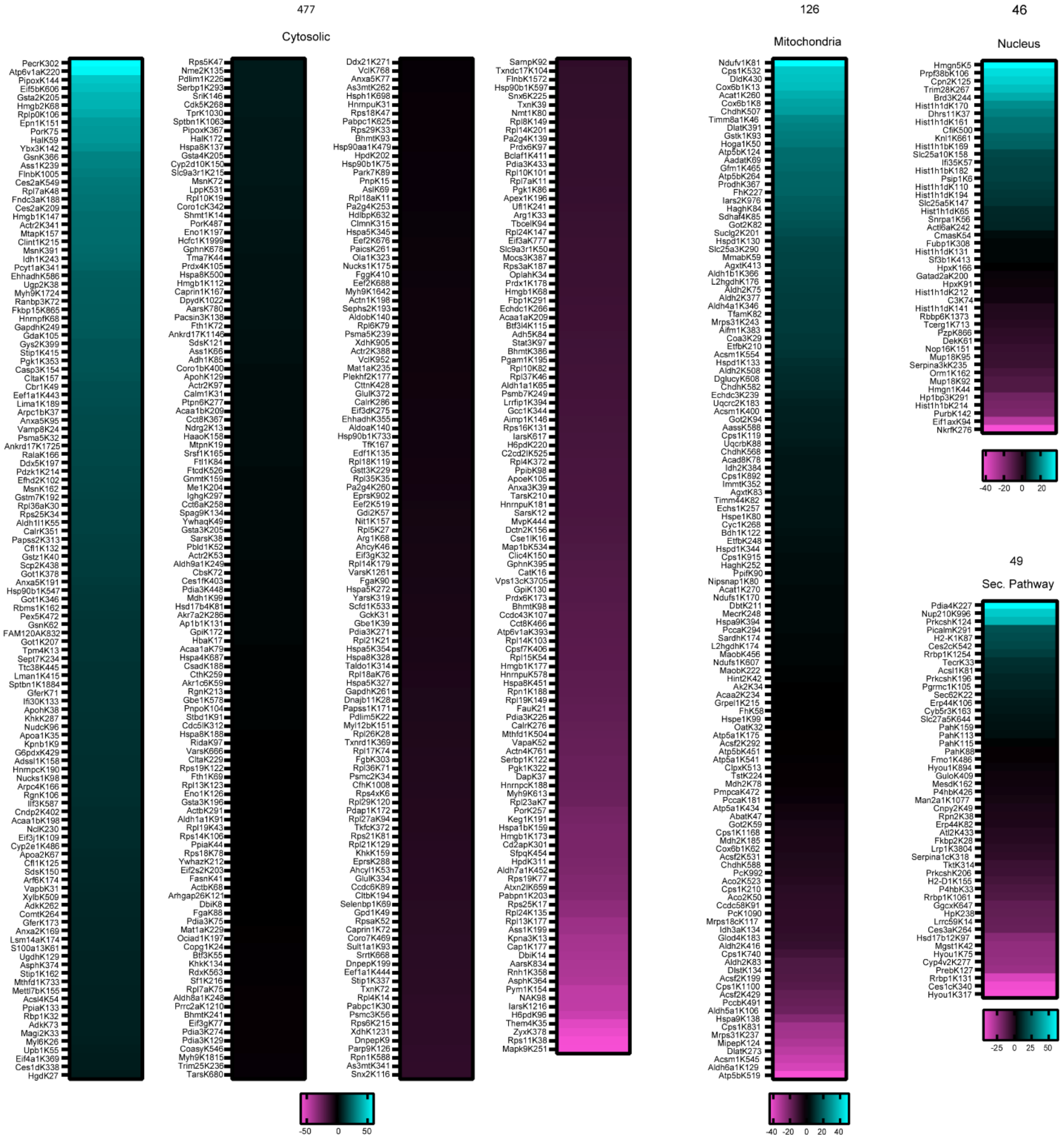

477
Cytosolic
-50
0
50
126
Mitochondria
-40
-20
0
20
40
46
Nucleus
-40
-20
0
20
49
Sec. Pathway
-25
0
25
50

Supplementary Figure 4. Heat-maps showing the profile of the 698 acetylation sites among the cytosolic, mitochondria, nucleus, and secretory pathway fractions found in SLC13A5 sTg mice, as compared to SLC25A1 sTg mice. Mice were 2 month-old when analyzed.

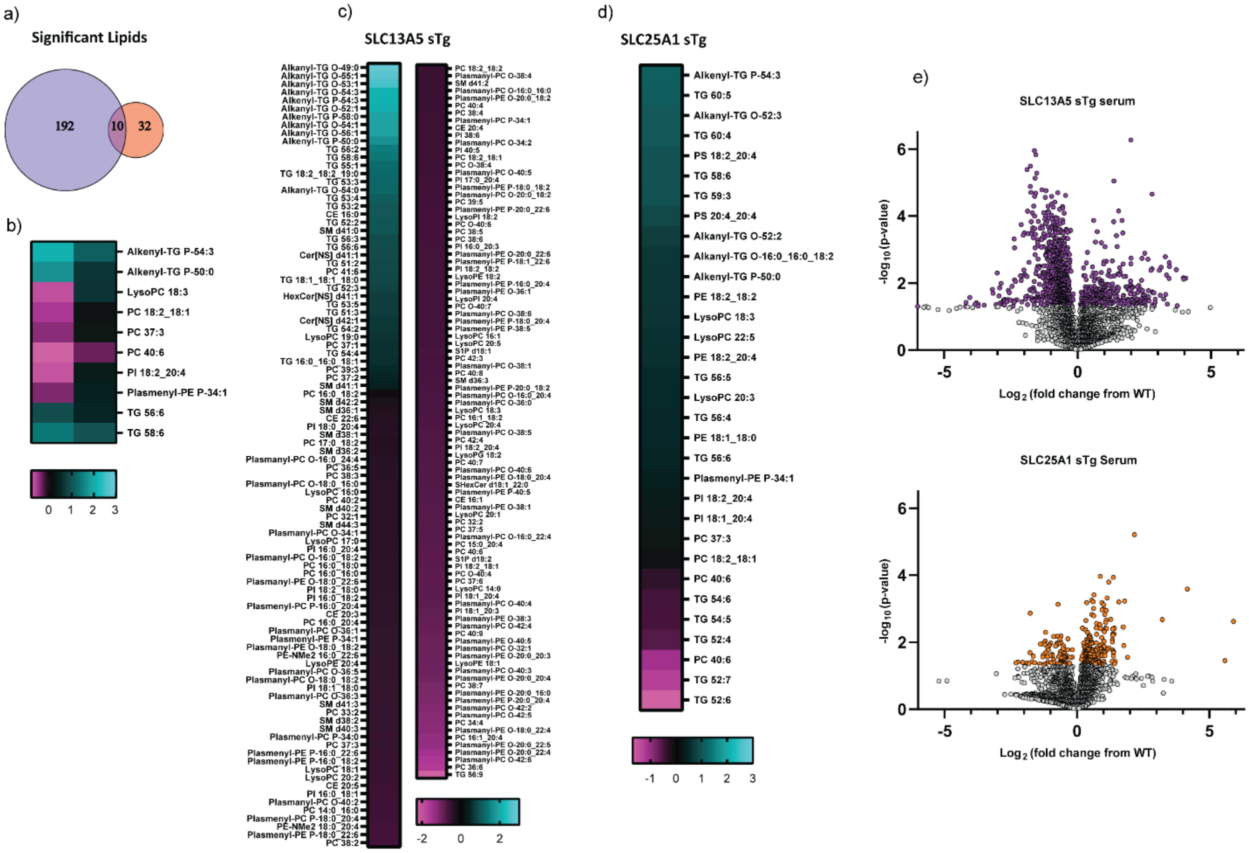
a)
Significant Lipids
192
10
32
b)
Alkenyl-TG P-54:3
Alkenyl-TG P-50:0
LysoPC 18:3
PC 18:2_18:1
PC 37:3
PC 40:6
PI 18:2_20:4
Plasmenyl-PE P-34:1
TG 56:6
TG 58:6
0 1 2 3
c)
SLC13A5 sTg
-2 0 2
d)
SLC25A1 sTg
Alkenyl-TG P-54:3
TG 60:5
Alkanyl-TG O-52:3
TG 60:4
PS 18:2_20:4
TG 58:6
TG 59:3
PS 20:4_20:4
Alkanyl-TG O-52:2
Alkanyl-TG O-16:0_16:0_18:2
Alkenyl-TG P-50:0
PE 18:2_18:2
LysoPC 18:3
LysoPC 22:5
PE 18:2_20:4
TG 56:5
LysoPC 20:3
TG 56:4
PE 18:1_18:0
TG 56:6
Plasmenyl-PE P-34:1
PI 18:2_20:4
PI 18:1_20:4
PC 37:3
PC 18:2_18:1
PC 40:6
TG 54:6
TG 54:5
TG 52:4
PC 40:6
TG 52:7
TG 52:6
-1 0 1 2 3
e)
SLC13A5 sTg serum
-log10(p-value)
Log2 (fold change from WT)
SLC25A1 sTg Serum
-log10(p-value)
Log2 (fold change from WT)

Supplementary Figure 5. SLC13A5 sTg and SLC25A1 sTg mice display different lipid adaptation in the serum.

(a) Venn diagram showing overlap between SLC13A5 sTg (purple) and SLC25A1 sTg (orange) mice across statistically significant lipids (n=5 male mice per group).

(b) Heat-map showing the expression profile of dysregulated lipids (total of 10) found in SLC13A5 sTg and SLC25A1 sTg mice, as compared to WT littermates.

(c) Heat-map showing the expression profile of dysregulated lipids (total of 192) found in SLC13A5 sTg mice, as compared to WT littermates.

(d) Heat-map showing the expression profile of dysregulated lipids (total of 32) found in SLC25A1 sTg mice, as compared to WT littermates.

(e) Volcano plots displaying all quantified lipids in SLC13A5 sTg and SLC25A1 sTg blood serum, as compared to age-matched WT littermates. Statistically significant species are highlighted in purple (for SLC13A5 sTg) and orange (for SLC25A1 sTg). $P<0.05$ via Fisher's method with all other species in grey.

Mice were 2 month-old when analyzed.

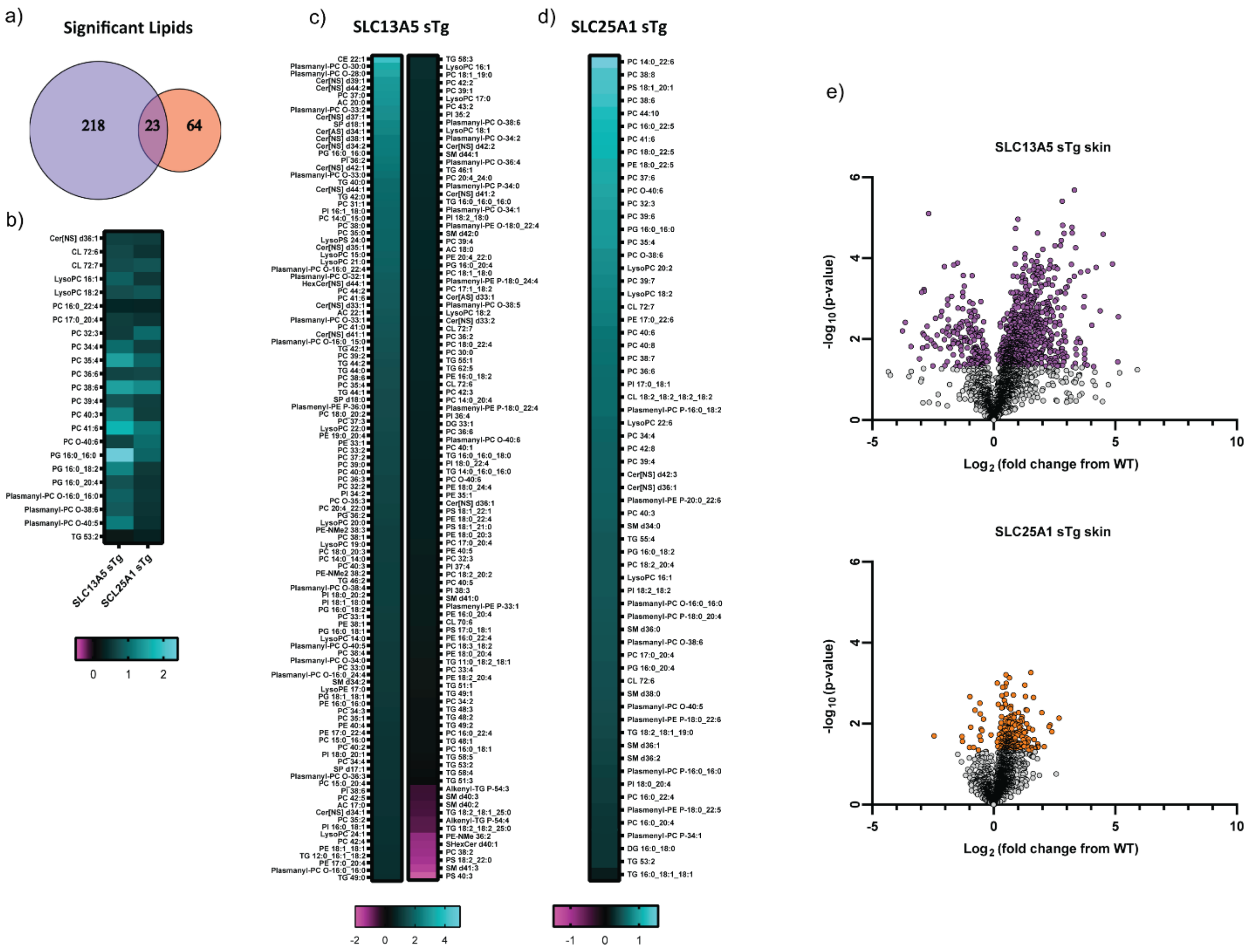
a)
Significant Lipids
218
23
64
b)
SLC13A5 sTg
SCL25A1 sTg
0
1
2
c)
SLC13A5 sTg
-2
0
2
4
d)
SLC25A1 sTg
-1
0
1
e)
SLC13A5 sTg skin
SLC25A1 sTg skin
-log$_{10}$ (p-value)
Log$_2$ (fold change from WT)
-5
0
5
10
0
2
4
6

Supplementary Figure 6. SLC13A5 sTg and SLC25A1 sTg mice display different lipid adaptation in the skin.

(a) Venn diagram showing overlap between SLC13A5 sTg (purple) and SLC25A1 sTg (orange) mice across statistically significant lipids (n=5 male mice per group).

(b) Heat-map showing the expression profile of dysregulated lipids (total of 23) found in SLC13A5 sTg and SLC25A1 sTg mice, as compared to WT littermates.

(c) Heat-map showing the expression profile of dysregulated lipids (total of 218) found in SLC13A5 sTg mice, as compared to WT littermates.

(d) Heat-map showing the expression profile of dysregulated lipids (total of 64) found in SLC25A1 sTg mice, as compared to WT littermates.

(e) Volcano plots displaying all quantified lipids in SLC13A5 sTg and SLC25A1 sTg skin tissue, as compared to age-matched WT littermates. Statistically significant species are highlighted in purple (for SLC13A5 sTg) and orange (for SLC25A1 sTg). $P<0.05$ via Fisher's method with all other species in grey.

Mice were 2 month-old when analyzed.

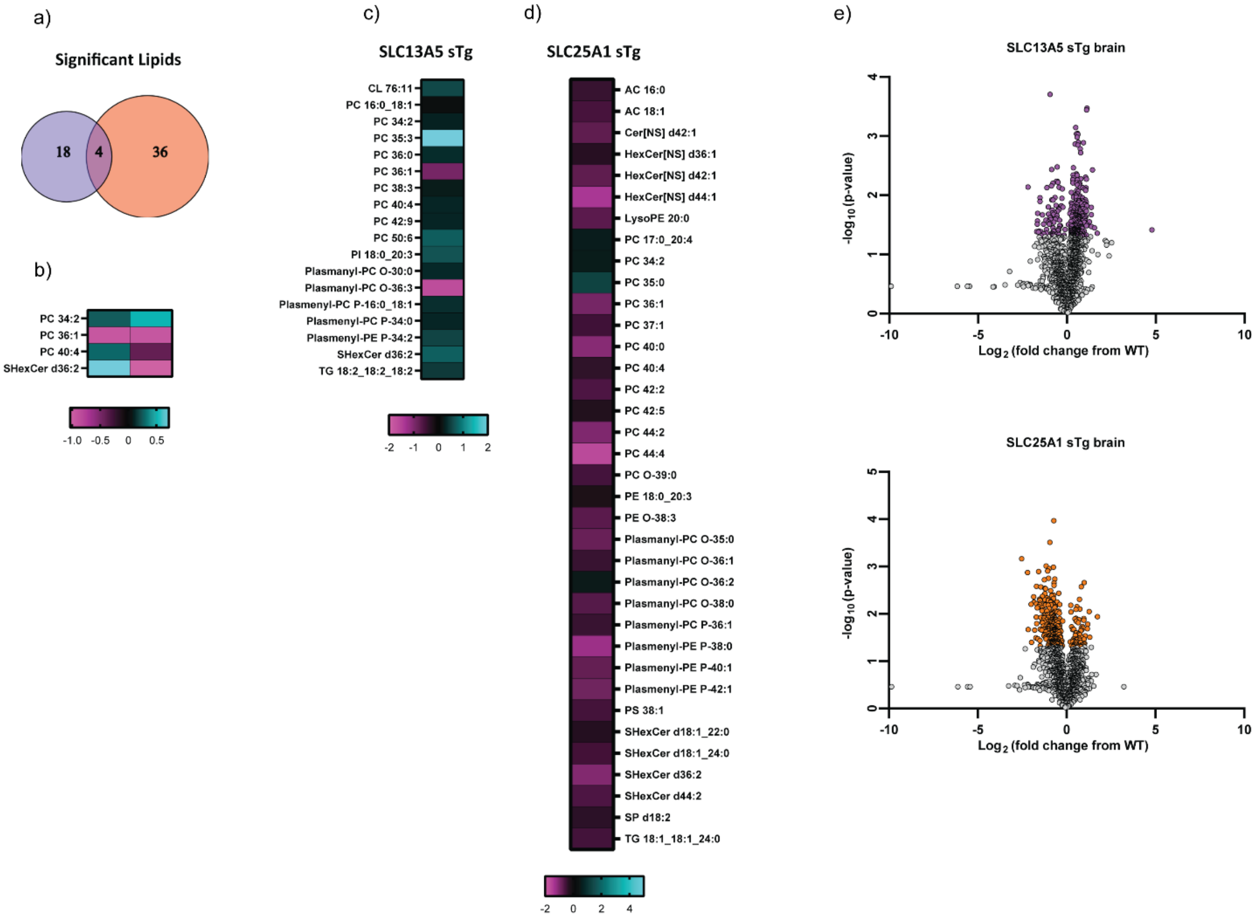
a)
Significant Lipids
18
4
36
b)
PC 34:2
PC 36:1
PC 40:4
SHexCer d36:2
-1.0 -0.5 0 0.5
c)
SLC13A5 sTg
CL 76:11
PC 16:0_18:1
PC 34:2
PC 35:3
PC 36:0
PC 36:1
PC 38:3
PC 40:4
PC 42:9
PC 50:6
PI 18:0_20:3
Plasmanyl-PC O-30:0
Plasmanyl-PC O-36:3
Plasmenyl-PC P-16:0_18:1
Plasmenyl-PC P-34:0
Plasmenyl-PE P-34:2
SHexCer d36:2
TG 18:2_18:2_18:2
-2 -1 0 1 2
d)
SLC25A1 sTg
AC 16:0
AC 18:1
Cer[NS] d42:1
HexCer[NS] d36:1
HexCer[NS] d42:1
HexCer[NS] d44:1
LysoPE 20:0
PC 17:0_20:4
PC 34:2
PC 35:0
PC 36:1
PC 37:1
PC 40:0
PC 40:4
PC 42:2
PC 42:5
PC 44:2
PC 44:4
PC O-39:0
PE 18:0_20:3
PE O-38:3
Plasmanyl-PC O-35:0
Plasmanyl-PC O-36:1
Plasmanyl-PC O-36:2
Plasmanyl-PC O-38:0
Plasmenyl-PC P-36:1
Plasmenyl-PE P-38:0
Plasmenyl-PE P-40:1
Plasmenyl-PE P-42:1
PS 38:1
SHexCer d18:1_22:0
SHexCer d18:1_24:0
SHexCer d36:2
SHexCer d44:2
SP d18:2
TG 18:1_18:1_24:0
-2 0 2 4
e)
SLC13A5 sTg brain
-log10(p-value)
Log2 (fold change from WT)
-10 -5 0 5 10
0 1 2 3 4
SLC25A1 sTg brain
-log10(p-value)
Log2 (fold change from WT)
-10 -5 0 5 10
0 1 2 3 4 5

Supplementary Figure 7. SLC13A5 sTg and SLC25A1 sTg mice display different lipid adaptation in the brain.

(a) Venn diagram showing overlap between SLC13A5 sTg (purple) and SLC25A1 sTg (orange) mice across statistically significant lipids (n=5 male mice per group).

(b) Heat-map showing the expression profile of dysregulated lipids (total of 4) found in SLC13A5 sTg and SLC25A1 sTg mice, as compared to WT littermates.

(c) Heat-map showing the expression profile of dysregulated lipids (total of 18) found in SLC13A5 sTg mice, as compared to WT littermates.

(d) Heat-map showing the expression profile of dysregulated lipids (total of 36) found in SLC25A1 sTg mice, as compared to WT littermates.

(e) Volcano plots displaying all quantified lipids in SLC13A5 sTg and SLC25A1 sTg brain, as compared to age-matched WT littermates. Statistically significant species are highlighted in purple (for SLC13A5 sTg) and orange (for SLC25A1 sTg). $P<0.05$ via Fisher's method with all other species in grey.

Mice were 2 month-old when analyzed.

a)

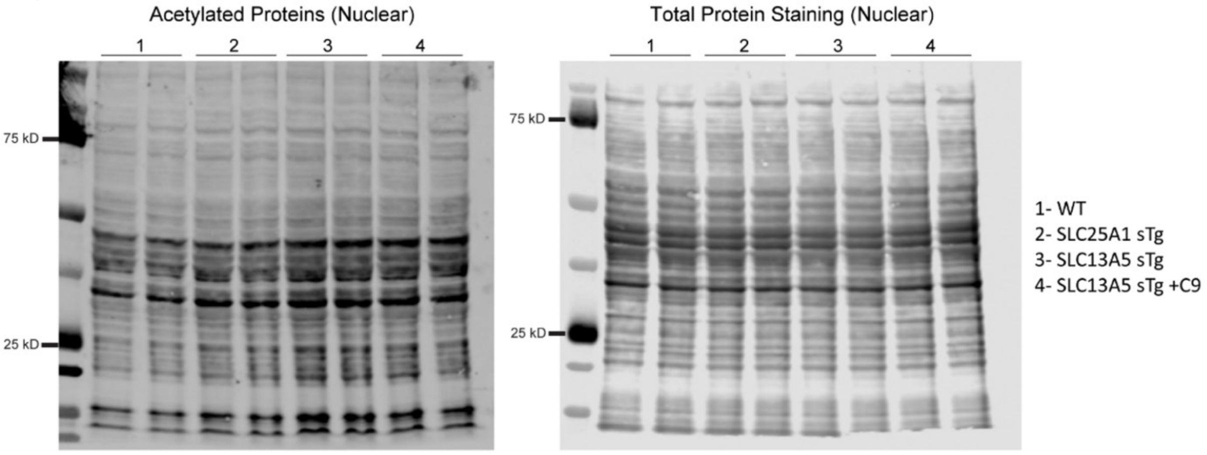
Acetylated Proteins (Nuclear)
Total Protein Staining (Nuclear)
1
2
3
4
75 kD
25 kD
1- WT
2- SLC25A1 sTg
3- SLC13A5 sTg
4- SLC13A5 sTg +C9

b)

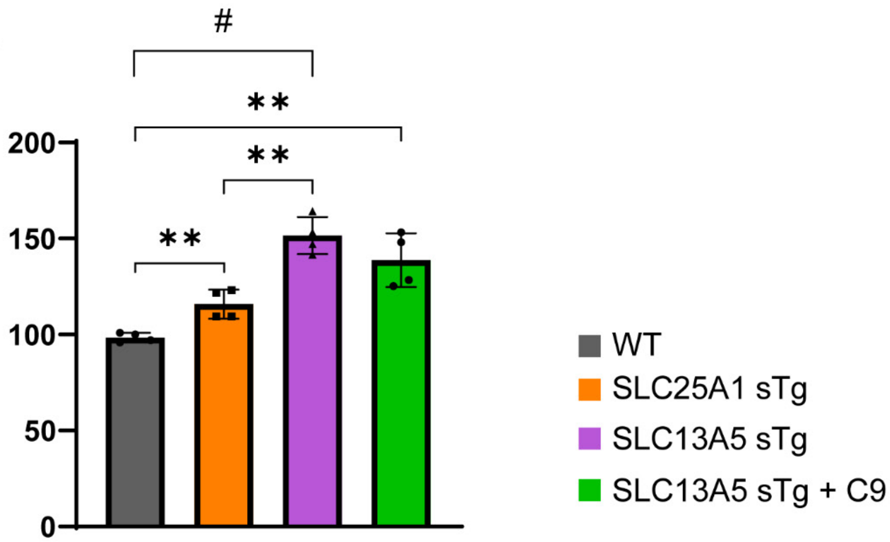
#
**
**
**
200
150
100
50
0
WT
SLC25A1 sTg
SLC13A5 sTg
SLC13A5 sTg + C9

c)

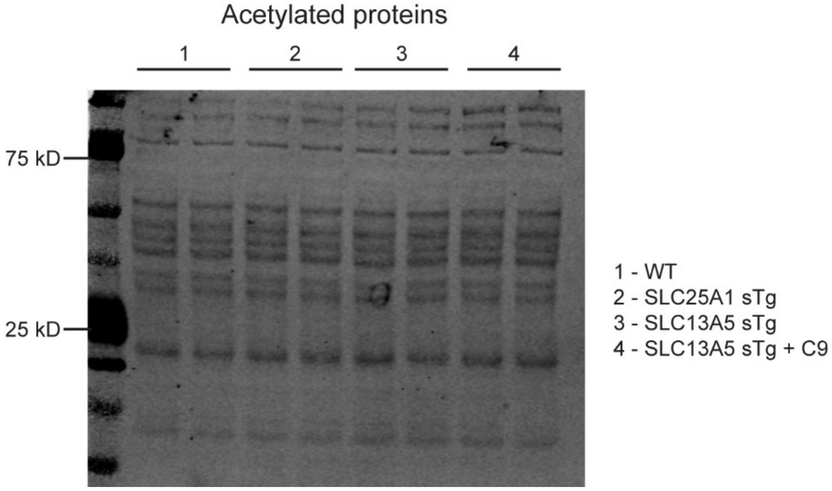
Acetylated proteins
1
2
3
4
75 kD
25 kD
1 - WT
2 - SLC25A1 sTg
3 - SLC13A5 sTg
4 - SLC13A5 sTg + C9

Supplementary Figure 8. The increased acetylation of the nucleus in SLC13A5 sTg mice is not rescued by C9.

(a-b) Western blot showing the profile of Nε-lysine acetylation in the nucleus isolated from the liver. Representative Western blot with total protein staining (a) and quantification (b) are shown. *C9*, Compound 9.

(c) Western blot showing the profile of Nε-lysine acetylation in the total liver lysate. *C9*, Compound 9.

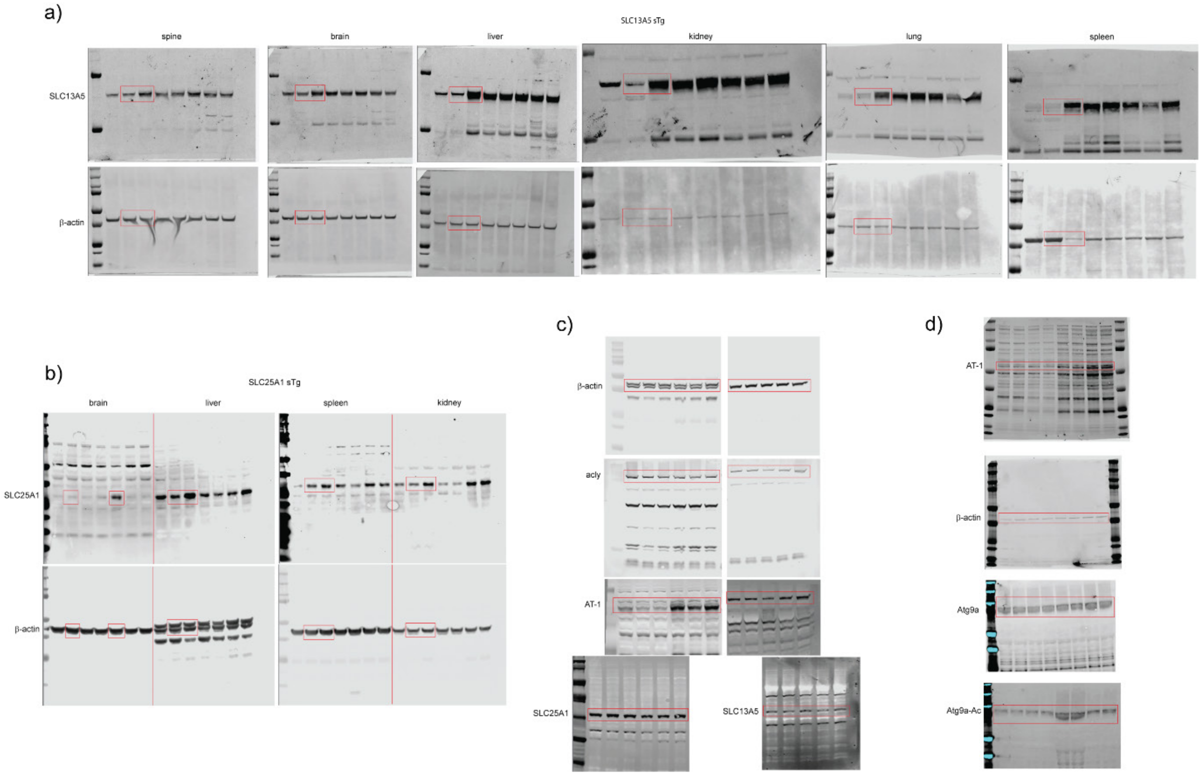

Supplementary Figure 9. Uncropped Western blots from Figure 1J (panel a and b), Figure 7b (panel c), Figure 7c and 7e (panel d).
