# Supplementary material for: The citrate transporters SLC13A5 and SLC25A1 elicit different metabolic responses and phenotypes in the mouse
Source: Commun Biol. 2023 Sep 9;6:926. doi: 10.1038/s42003-023-05311-1 (PMC10492862; doi:10.1038/s42003-023-05311-1)
Supplement: Supplementary file 3 — Description of Additional Supplementary Files [file 42003_2023_5311_MOESM3_ESM.pdf]

Description of Additional Supplementary Files

File name: Supplementary Data 1
Description: The source data behind the graphs and charts in the paper.

File name: Supplementary Movie 1
Description: Phenotypic correction of the SLC13A5 phenotype by the ATase inhibitor, compound 9.
